# Kirk R Smith: a life’s work on improving air quality

**DOI:** 10.2471/BLT.20.272054

**Published:** 2020-08-01

**Authors:** Heather Adair-Rohani

**Affiliations:** a Department of Environment, Climate Change and Health, World Health Organization, avenue Appia 20, 1211 Geneva 27, Switzerland.

Many people strive to improve the world around them, but few have impacted millions of lives the way the late Professor Kirk Robert Smith has. From championing the right of the poorest populations to have a clean stove for cooking, to building knowledge and inspiring students, researchers and policy-makers to address environmental health risks (particularly air pollution and climate change), Smith was a true public health champion and leader. 

Smith started as a researcher at the East-West Center in Hawaii, United States of America (USA), later serving as an environmental health professor and director of the Global Health and Environment programme at the School of Public Health, University of California, Berkeley, USA. He was also one of the founders and director of the Collaborative Clean Air Policy Centre in New Delhi, India.

Smith started his career focused on nuclear risk management, but after spending time in India and Nepal, he identified a much greater risk to public health: the smoke or air pollution generated by simply preparing a hot meal, or staying warm on a cold night, a daily risk of necessity for the poorest populations.

Recognizing the gravity and potential magnitude of this problem, Smith’s first instinct as a scientist was to measure exposure and quantify related health risk. In 1981, Smith and a group of Indian researchers measured a woman’s exposure to household air pollution for the first time, creating a new field of public health science. Over the next decades, Smith worked to quantify the health and other effects of polluting home energy use and to identify practical solutions^1^^–^^3^ to address the health crisis caused by household air pollution.

Smith’s pioneer work on air quality monitoring, technological interventions, quantification of health risk and implementation science, as well as his leadership, have been pivotal in shaping and building the World Health Organization’s (WHO) air quality and health programmes.

Working with colleagues at all levels of WHO, Smith’s influence was global. He played a main role in numerous WHO advisory groups and was instrumental in the preparation of WHO air quality guidelines, in particular those for indoor air quality related to household fuel combustion, and their worldwide promotion.^4^ He contributed to many WHO publications, the first in 1985, where he introduced the health risk of household (indoor) air pollution to the world stage.^5^ His calls to support WHO were fundamental in elevating the health sector’s role in improving air quality to protect public health.

One of his greatest research achievements was the RESPIRE (Randomized Exposure Study of Pollution Indoors and Respiratory Effects) trial, famous for its innovative experimental study design and robust quantification of childhood pneumonia risk from household air pollution.^6^ The trial provided the first empirical evidence and clarity as to just how clean cooking solutions need to be to protect health. In addition, this work helped to build the understanding of how much this indoor pollution was leaking outdoors and contributing to ambient air pollution exposure, further strengthening the argument for accelerating the transition to clean cooking.

Smith’s contribution to science and education was well recognized. He was elected to the National Academy of Sciences in 1997, received visiting distinguished professorships at top universities in China, India and Mongolia and won numerous individual awards, including in 2009 the Heinz Award with Special Focus on the Environment, and the 2012 Tyler Prize for Environmental Achievement. In 2007, Smith shared the Nobel Prize with the other members of the Intergovernmental Panel on Climate Change and former USA Vice President Al Gore.

One of Smith’s most lasting achievements was the community he built. Despite the hours spent in the field, teaching or researching, Smith always found time to educate, train and most importantly, mentor those interested in joining his public health mission. He has recruited and connected actors in the field and across the world. 

Smith challenged countries, such as India, to think beyond the simple solutions like improved cookstoves and for more equitable and healthy solutions, such as liquified petroleum gas or electric induction cookers.^7^ He pushed everyone to understand and appreciate the injustices of settling for sub-par cooking solutions for the poorest populations. Smith dedicated his life’s work to the change taking place today, as clean cooking is recognized as a critical step towards achieving sustainable development. 

Smith will be remembered in history as the father of clean cooking. The global public health community has lost a tireless mentor, advocate, leader and friend, but his memory, work and inspiration will live on.
